# Guilt Proneness and Shame Proneness Differentially Relate to Sex Disparity in Depression Among Adolescents in China: A Longitudinal Serial Mediation Analysis

**DOI:** 10.1007/s10964-026-02403-z

**Published:** 2026-08-26

**Authors:** Chengli Huang, Yanqiu Yu, Hongsheng Yang, Mengni Du, Liping Yang, Hongtao Shao, Tingjun Ye, Qun Zhu, Deborah Baofeng Wang, Joseph T.F. Lau

**Affiliations:** 1 School of Mental Health, Wenzhou Medical University, Wenzhou, China; 2 School of Public Health, Fudan University, Shanghai, China; 3 The Affiliated Wenzhou Kangning Hospital, Wenzhou Medical University, Wenzhou, China; 4 Teaching and Research Center, Bureau of Education, Taizhou, China; 5 Yiwu Mental Health Center, Yiwu, China; 6 Center for Health Behaviors Research, Jockey Club School of Public Health and Primary Care, The Chinese University of Hong Kong, Hong Kong SAR Hong Kong, China

**Keywords:** Sex, Shame, Guilt, Emotion regulation, Depressive symptoms

## Abstract

While sex disparities in adolescent depression are well-documented, how distinct self-conscious emotions divergently impact this gap remains incompletely understood. Using a three-wave longitudinal design, Chinese adolescents (*N* = 2,915; *M*_*age*_ = 16.99, *SD* = 1.33; 40.07% female) completed measures of shame, guilt, maladaptive cognitive emotion regulation, and depression over 12 months. Females exhibited higher shame, guilt, maladaptive cognitive emotion regulation, and depressive symptoms. Crucially, shame mediated the sex disparity by increasing depressive risk, whereas guilt was not a significant mediator. Furthermore, maladaptive cognitive emotion regulation serially mediated the risk pathway (Sex → Shame → Maladaptive cognitive emotion regulation → Depressive symptoms). This demonstrates that female depressive vulnerability is driven by a specific shame-induced regulatory failure, highlighting the need for interventions dismantling shame.

**Supplementary Information:**

The online version contains supplementary material available at https://doi.org/10.1007/s10964-026-02403-z.

## Introduction

Adolescent depression is a pressing global public health challenge that carries severe long-term consequences, including suicidal ideation and risk behaviors that persist into adulthood (Collaborators, 2022; Wang et al., 2024). Over the past decade, prevalence rates have risen sharply worldwide, with females exhibiting a markedly higher risk than males (Keyes & Platt, 2024; Thapar et al., 2022). Although factors such as self-conscious emotions and cognitive emotion regulation are known to influence depression, the longitudinal mechanisms explaining this pronounced sex disparity remain largely underexplored. To address this research gap, the present study investigates whether sex differences in shame, guilt, and maladaptive cognitive emotion regulation explain the higher prevalence of depressive symptoms in female adolescents in China.

### Sex Disparity in Depression

Sex disparity in mental health problems, starting from childhood and adolescence, has been well documented. Females exhibit consistently higher prevalence of depression than males across lifespan (Parker & Brotchie, 2010). This sex disparity typically emerges in early-to-mid adolescence (ages 13–15) (Girgus & Yang, 2015), and adolescent females are more likely than males to undergo elevating depressive trajectories extending into adulthood (Shore et al., 2018; Weavers et al., 2021). Sex disparities in consequences and factors of adolescent depression are also evident. Females with depressive disorders consistently account for a greater burden of diseases than males, such as higher years lived with disability (Ferrari et al., 2013). The psychosocial consequences of untreated depression in adolescent girls are profound, frequently manifesting as increases in health-risk behaviors (e.g., higher prevalence of unplanned pregnancy) that exacerbate long-term socioeconomic disadvantages (Thapar et al., 2012). Adolescent depressive symptomatology is associated with increased odds of school dropout among females, but not among males (Needham, 2009). Together, sex difference in adolescent depression is not merely a transient developmental phenomenon, but a critical public health issue leading to long-term and inequitable personal and socioeconomic disparities. To inform effective interventions, longitudinal research is required to uncovering the specific mediating mechanisms.

### Self-Conscious Emotions in Sex Disparity in Depression

Researchers have begun to propose various models to explain the sex disparity in mental distress, emphasizing differential stress exposure and maladaptive behaviors (For a review, see: Girgus & Yang, 2015). Yet, very few studies have investigated such mechanisms in the context of core developmental priority among adolescents, such as the formation of self and identity (Sebastian et al., 2008). Self-conscious emotions play profound roles in adolescent development. Unlike basic emotions, these effects are deeply rooted in self-evaluation and self-reflection within both interpersonal and intrapersonal contexts (Sznycer, 2019). Besides, self-conscious emotions such as guilt and shame are simultaneously associated with sex (Else-Quest et al., 2012) and depressive symptoms (Kim et al., 2011). Therefore, they may partially explain the sex difference in adolescent depressive symptoms.

Guilt and shame are negatively valanced self-conscious evaluative emotions that individuals perceive failure of the self when they are unable to meet important social standards, rules, or goals (Lewis, 2018). The early manifestations of guilt and shame emerge in toddlerhood (Kochanska et al., 2002) and are refined during adolescence (Gavazzi et al., 2011). Guilt and shame are related but distinct constructs. Specifically, guilt is defined as the reaction of one’s internalized conscience to a breach of one’s personal standard, whereas shame is defined as an affective reaction that follows public exposure (and disapproval) to impropriety or shortcoming (Tangney & Dearing, 2003). Guilt arises from a negative evaluation of a “specific” self (e.g., “I did a bad thing”), whereas shame entails a negative evaluation of the “global” self (“I am a bad person”) (Lewis, 2018). Critically, the difference between shame and guilt also extends to depressive symptoms (Tilghman-Osborne et al., 2008). Meta-analyses consistently report a stronger association between shame and depressive symptoms than guilt among children, adolescents, and adults (Kim et al., 2011; Muris & Meesters, 2014). Notably, when the variance attributable to shame is statistically controlled, the association between guilt and depressive symptoms diminishes or even reverses (Orth et al., 2010; Webb et al., 2007). Similarly, individual proneness to shame and guilt were positively and negatively associated with expressive suppression, respectively, which in turn positively linked to depressive symptoms (Liw et al., 2022).

Guilt and shame proneness also display sex differences, with females showing higher levels of both (Else-Quest et al., 2012; Orth et al., 2010). This disparity aligns with the well-established framework that females typically construct their identities with a stronger relational-interdependent orientation (Cyranowski et al., 2000). Because shame and guilt inherently signal perturbations within interpersonal bonds (Sznycer, 2019), these emotions theoretically trigger stronger self-evaluative distress in females, leading to stronger associations with depressive symptoms. For instance, specific facets like body shame have already been identified as a theoretically crucial factors in explaining sex differences in depression (Hyde et al., 2008). By extension, guilt and shame function as broader underlying mechanisms linking sex to adolescent depressive symptoms (Kim et al., 2011). However, empirical evidence regarding whether these emotional traits longitudinally explain the sex disparity in depression remains limited.

### Maladaptive Cognitive Emotion Regulation in Sex Disparity in Depression

Maladaptive cognitive emotion regulation refers to cognitive strategies (e.g., rumination, catastrophizing, self-blame, and other-blame) that individuals deploy in response to threatening or stressful events. These negatively focused strategies are robustly linked to the development and maintenance of depression (Hyde & Mezulis, 2020). A well-documented sex disparity exists in the use of these strategies. Female (vs. male) adolescents are more likely to evoke maladaptive cognitive emotion regulation because gendered socialization patterns encourage females to utilize emotional internalization strategies (Nolen-Hoeksema, 2012). Moreover, sex differences in maladaptive cognitive emotion regulation also reflect broader differences in response style, whereby females are more likely to engage in self-focused processing and repetitive negative thinking in response to stress, increasing their reliance on ruminative and self-blaming strategies (Johnson & Whisman, 2013; Nolen-Hoeksema et al., 2008). While maladaptive cognitive emotion regulation is known to mediate the relationship between sex and depression (Hyde & Mezulis, 2020; Nolen-Hoeksema, 2012), the specific affective triggers of these cognitive strategies require deeper conceptual integration.

Conceptually, the deployment of maladaptive cognitive emotion regulation is intimately tied to the distinct functional trajectories of shame and guilt. Specifically, guilt is typically characterized by reparative motivation and adaptive behavioral correction, whereas shame tends to elicit maladaptive withdrawal-oriented responses, such as social withdrawal (Cohen et al., 2011). In this vein, reduced use of maladaptive cognitive emotion regulation strategies was linked to guilt, whereas greater reliance on such strategies was positively associated with shame (Estévez et al., 2024; Szentágotai-Tătar & Miu, 2016). Together, the existing literature implies a potential sequential mechanism underlying the sex disparity in adolescent depression via guilt/shame and subsequent maladaptive cognitive emotion regulation. In fact, cross-sectional evidence further suggests the adverse effect of shame on depressive symptoms was substantially mediated by maladaptive cognitive emotion regulation (e.g. rumination; Cheung et al., 2004), whereas comparable mediation effects are typically absent for guilt (Orth et al., 2006). However, longitudinal evidence directly examining prospective associations among sex, guilt/shame proneness, maladaptive cognitive emotion regulation, and depressive symptoms via a serial mediation structure remains scarce.

## Current Study

Although adolescent females generally exhibit higher levels of self-conscious emotions, maladaptive cognitive emotion regulation, and depressive symptoms, it remains unclear how shame and guilt differentially influence maladaptive cognitive emotion regulation over time to explain the sex disparity in depressive symptoms. Based on the above literature review, shame is more likely to be maintained and amplified through maladaptive cognitive emotion regulation, thereby increasing vulnerability to depressive symptoms; in contrast, guilt is less likely to be processed via maladaptive cognitive emotion regulation, thereby conferring relative protection against depressive symptoms. To address this research gap, the present longitudinal study aimed to examine the prospective pathways linking sex to depressive symptoms through shame and guilt proneness and subsequent maladaptive cognitive emotion regulation. Based on the theoretical rationales, the present study expected to find significant sex differences, with females reporting higher levels of self-conscious emotions, maladaptive regulation, and depressive symptoms (Hypothesis 1). Furthermore, these affective and cognitive factors would mediate the relationship between sex and depressive symptoms (Sex → Self-conscious emotions/Maladaptive cognitive emotion regulation → Depressive symptoms; Hypothesis 2). Crucially, this study further hypothesized a serial indirect pathway linking sex to depressive symptoms, driven sequentially by self-conscious emotions and maladaptive cognitive emotion regulation (Sex → Self-conscious emotions → Maladaptive cognitive emotion regulation → Depressive symptoms; Hypothesis 3). Finally, because the functional outcomes of these emotions diverge, the current study further expected this cognitive regulatory mechanism to play a differential role—amplifying the pathway from shame to depressive symptoms, while having minimal or countervailing relevance for guilt—thereby resulting in a combination of mediation and suppression effects (Hypothesis 4).

## Methods

### Participants

A 3-wave longitudinal survey with 6-month intervals was conducted in four schools from June 2024 to June 2025 located in Taizhou, Zhejiang Province, China. All grade 1 and 2 students of the junior middle schools (typically aged 14–16 years), grade 1 students of the senior middle school (typically aged 17–19 years), grade 1 and 2 students of another senior middle school, and grade 1 and 2 students of a vocational high school (typically aged 17–19 years) were invited to join the study. In the classroom, the fieldworkers provided a brief explanation of the purpose of the study to the students and informed them that the return of the completed questionnaire implied informed consent to participate in the study. The participants were reminded that participation was totally voluntary, and they could refuse to join the study or quit anytime without facing any negative consequences. This information was also printed on the cover page of the questionnaire. The students then self-administered the structured questionnaire in the absence of teachers and without discussing with each other. The questionnaire took about 15 min to complete. Furthermore, parents were notified about the study and given the option to decline their child’s participation by sending a note to the teachers, in which case the child would not be invited to participate in the study. Such a procedure has been used in other studies (Shao et al., 2026). No incentives were provided to the participants. No personal identification information was included in the database. This study was approved by the Research Ethics Committee of Wenzhou Medical University (No. KNLL-2022007).

A total of 3,734 students completed the baseline survey (T1). Data obtained from 2,915 (78.07%) students were matched by their grade, class, and seat number for the three waves, which was used for data analysis. An attrition analysis was conducted by comparing participants who completed all three waves (*n* = 2,915) with those who dropped out at any subsequent wave (*n* = 819) in baseline variables. Results indicated that completers and dropouts differed significantly in age, depressive symptoms, catastrophizing, guilt proneness in negative behavior evaluation, guilt proneness in repair action, school type, and single parenthood. Importantly, all observed effect sizes were generally small (Cohen’s *d* ≦ 0.12; Cramer’s V ≦ 0.10), except for age (Cohen’s *d* = 0.31), suggesting limited evidence of substantial attrition bias. Detailed attrition analyses are presented in Appendix (Section Attrition Analyses).

### Measures

#### Socio-Demographic Information

Such data included age, sex, type of school attended (junior middle school, senior middle school, or vocational high school), single parenthood (no or yes), living arrangement (co-residing with both parents, father only, mother only, or neither), father’s and mother’s educational level (junior middle school or below, senior high school or equivalent, or college and above), and self-reported family financial status (very poor, poor, average, rich, very rich).

#### Assessment of Shame and Guilt Proneness

Shame and guilt proneness were measured using the 16-item Guilt and Shame Proneness Scale (GASP; Cohen et al., 2011), which has been validated among Chinese university students with acceptable psychometric properties (Wang et al., 2016), and have subsequently been used in research targeting secondary students (Tian, 2024). The GASP comprises two subscales for guilt proneness that assess negative behavior-evaluations (GP-NBE) and repair action tendencies (GP-RA). Two similar shame proneness subscales were used to assess the negative self-evaluations (SP-NSE) and withdrawal action tendencies (SP-WA)^1^. Each subscale comprises four items. The participants read a description of a personal transgression and indicated the likelihood that they would respond in the way described with a 7-point Likert scale (1 = very unlikely to 7 = very likely). A sample item is “After realizing you have received too much change at a store, you decide to keep it because the salesclerk doesn’t notice. What is the likelihood that you would feel uncomfortable about keeping the money?” Each subscale score was computed as the mean of its four items, with higher scores indicating higher levels of the two respective aspects of guilt and shame proneness. To ensure the construct validity of GASP for the early adolescent sample, Confirmatory Factor Analyses (CFA) were conducted. The four-factor model for GASP demonstrated acceptable overall fit (χ² = 921.88, *df* = 98, CFI = 0.901, TLI = 0.878, RMSEA = 0.056, SRMR = 0.048). The Cronbach’s alphas of three subscales were acceptable, ranging from 0.70 to 0.74 but was 0.52 for the SP-WA subscale. This relatively low internal consistency is consistent with findings from the original GASP validation (α = 0.60; Cohen et al., 2011) and other studies (e.g., Tian, 2024; Wang et al., 2016; Veneziani et al., 2024). Given this low reliability, a sensitivity analysis excluding the SP-WA subscale was conducted (see Section Sensitivity Analysis).

#### Maladaptive Cognitive Emotional Regulation (MCER)

It was measured by using the three subscales—rumination, self-blame, and catastrophizing—of the short 18-item version of the Cognitive Emotion Regulation Questionnaire (CERQ-short; Garnefski & Kraaij, 2006), which has been used among Chinese university students with acceptable psychometric properties (Yu et al., 2024). Self-blame was assessed by the frequency of attributing the cause of negative experiences to oneself; rumination was measured by the rate of repeatedly thinking about the feelings and thoughts associated with past negative events; catastrophizing was evaluated by the extent of exaggerating the terror and negative consequences of experienced events (Garnefski & Kraaij, 2006). Each subscale comprises two items, and the items were measured on a 5-point Likert scale (1 = almost never to 5 = always). A sample item is “I keep thinking about how terrible it is what I have experienced.” Subscale scores were obtained by summing up the item scores belonging to the respective subscale (ranging from 2 to 10). The higher the subscale score, the more often a specific emotional regulation strategy was used. To ensure the construct validity of MCER for the early adolescent sample, Confirmatory Factor Analyses (CFA) were conducted. The three-factor model for the MCER exhibited acceptable fit indices (*χ²* = 92.26, *df* = 6, CFI = 0.984, TLI = 0.961, RMSEA = 0.073, SRMR = 0.020). The Cronbach’s alphas of the subscales were acceptable, ranging from 0.80 to 0.93 at T1 and T2.

#### Depressive Symptoms

It was measured by the 9-item Patient Health Questionnaire (PHQ; Kroenke et al., 2001). Its Chinese version has been validated across gender and age groups in Chinese adolescents, showing acceptable psychometric properties (Leung et al., 2020). A sample item is “Feeling down, depressed, or hopeless.” The scale was assessed with a 4-point Likert scale (0 = Not at all to 3 = Nearly every day). Depressive symptoms were assessed by summing up all item scores, with higher scores indicating higher levels of depressive symptoms. Its overall score of between 0 and 27. In the present study, Cronbach’s alphas were 0.87, 0.89, and 0.89 at T1, T2, and T3, respectively.

### Statistical Analysis

Bivariate linear regression analysis was conducted to test the associations between the background factors and depressive symptoms at T1, identifying relevant covariates for inclusion in the subsequent structural equation model (SEM). Spearman correlation analysis was conducted to test the significance of the correlations among the key variables at relevant time points [i.e., guilt proneness (T1), shame proneness (T1), the three MCER variables of rumination, catastrophizing, self-blame (T2), and depressive symptoms (T3)], establishing the basic pattern of associations among the focal constructs. Independent sample t-tests were used to compare sex differences in the levels of such four key variables. Structural equation modeling (SEM), using the Maximum Likelihood Estimator, was conducted to test the mediation effect of guilt/shame proneness at T1 and MCER at T2 between sex and depressive symptoms at T3 (the independent variable, IV). MCER^2^ was created as a second-order latent variable defined by three first-order factors (rumination, self-blame, and catastrophizing), each constructed from two items of their respective original subscales (Yu et al., 2024). Standardized coefficients (*β*) were reported, satisfactory model fit indices were Comparative Fit Index (CFI) ≥ 0.90, Root Mean Square Error of Approximation (RMSEA) ≤ 0.08, and Standardized Root Mean Square Residual (SRMR) ≤ 0.08 (Kline, 2023). All analyses were conducted in R, with the exception of the Confirmatory Factor Analyses (CFA) and Measurement Invariance Test (MIT), which were performed in Mplus 8.3. Missing values were handled using Full Information Maximum Likelihood (FIML) procedure (Schafer & Graham, 2002). Statistical significance was defined as a two-tailed *p*-value < 0.05.

## Results

### Descriptive Statistics

The participants’ socio-demographic characteristics at baseline (T1) are shown in Table 1. About 60% of the participants were male; 9% living in a single parent family; 75% living with both parents. About 12% and 13% of the participants’ father and mother had attained post-secondary education. About 70% self-reported an average level of family income (low: 8.57%; high: 18.05%). At the three time-points, the pooled point prevalence of depressive symptoms (PHQ-9 ≥ 10) was 28.54%, 22.92%, and 26.38%, respectively.

**Table 1:** Socio-demographic characteristics measured at the baseline (*N* = 2,915)

	*N*	Percentage (%)
School type		
Junior middle school	692	23.74
Senior high school	807	27.68
Vocational high school	1,416	48.58
Sex		
Male	1,747	59.93
Female	1,168	40.07
Single parenthood		
No	2,474	84.87
Yes	274	9.40
Not reported	167	5.73
Living arrangement		
Co-residing with both parents	2,194	75.27
Co-residing with mother	302	1.36
Co-residing with father	141	4.84
Co-residing with neither	278	9.54
Father’s education level		
Elementary or lower	338	11.60
Middle school (Junior high)	1,312	45.01
High school (Senior high)	804	27.58
Post-secondary	155	5.32
Undergraduate college or higher	203	6.96
Not reported	103	3.53
Mother’s education level		
Elementary or lower	498	17.08
Middle school	1,265	43.40
High school	665	22.81
Post-secondary	165	5.66
Undergraduate college or higher	220	7.55
Not reported	102	3.50
Self-reported family financial status		
Very poor	47	1.61
Poor	203	6.96
Average	2,033	69.74
Rich	487	16.71
Very rich	39	1.34
Not clear	106	3.64
**Continuous variables**	**Mean**	**Standard deviation**
Age	16.99	1.33

### Gender Differences in the Key Variables Studied at Baseline (T1)

As Table 2 shown, females (versus males) showed significantly higher guilt proneness, higher shame proneness, stronger MCER, and higher level of depressive symptoms at T1, the effect sizes ranged from 0.11 to 0.60. Similar differences are found for comparisons made at T2 and T3. These findings provided support for Hypothesis 1.

**Table 2:** Levels of the key variables by sex at baseline (T1) (*N* = 2,915)

	Female			Male			*p*	Effect size (Cohen’s d)
	*N*	Mean	SD	*N*	Mean	SD		
GP-NBE	1,166	5.89	0.99	1,742	5.50	1.19	< 0.001	0.36
GP-RA	1,168	5.65	0.97	1,742	5.31	1.13	< 0.001	0.32
SP-NSE	1,167	5.23	1.18	1,742	4.63	1.23	< 0.001	0.50
SP-WA	1,167	4.12	1.07	1,742	3.47	1.10	< 0.001	0.60
Self-blame	1,167	4.63	2.09	1,731	4.38	1.94	< 0.001	0.13
Rumination	1,168	5.66	2.13	1,731	5.41	2.10	0.003	0.11
Catastrophizing	1,167	4.38	2.29	1,731	3.92	2.04	< 0.001	0.21
Depressive symptoms	1,167	8.28	5.08	1,740	6.92	4.90	< 0.001	0.27

Note. SD = Standard deviation. GP-NBE = Guilt Proneness-Negative Behavior Evaluation subscale; GP-RA = Guilt Proneness-Repair Action Subscale; SP-NSE = Shame Proneness-Negative Self Evaluation subscale; SP-WA = Shame Proneness-Repair Action Subscale. Effect size (Cohen’s *d*) represents the mean difference between female and male students *Mean*_*female*_ - *Mean*_*male*_. A positive *d* reflects a higher mean in the female sub-sample

### Associations Between the Background Factors and Depressive Symptoms at T1

Table 3 present the associations between the background factors and depressive symptoms at T1. Variables significantly associated with level of depressive symptoms included older age, female sex, studying in a senior high school (versus junior middle school), living in a single-parent household, residing only with the father or not with any parent (versus living with both parents), poor self-reported family financial status, and lower parental education.

**Table 3:** Associations between the background factors and depressive symptoms at T1

	B	SE	β	*p*
Age (years)	0.18	0.07	0.05	*0.012*
Sex				
Male	Ref			
Female	1.38	0.19	0.14	< 0.001
School type				
Junior middle school	Ref			
Senior high school	1.24	0.27	0.11	< 0.001
Vocational high school	0.20	0.23	0.02	0.398
Single parenthood				
No	Ref			
Yes	0.72	0.32	0.04	0.023
Living arrangement				
Co-residing with both parents	Ref			
Co-residing with mother	0.36	0.31	0.02	0.246
Co-residing with father	0.89	0.43	0.04	0.040
Co-residing with neither	0.98	0.32	0.06	0.002
Father’s education level				
Elementary or lower	Ref			
Middle school	− 0.80	0.28	− 0.08	0.004
High school	− 0.85	0.30	− 0.08	0.004
Post-secondary	-1.43	0.47	− 0.06	0.002
Undergraduate college or higher	− 0.17	0.42	− 0.01	0.681
Mother’s education level				
Elementary or lower	Ref			
Middle school	− 0.92	0.25	− 0.09	< 0.001
High school	-1.02	0.28	− 0.09	< 0.001
Post-secondary	-1.33	0.44	− 0.06	0.002
Undergraduate college or higher	0.33	0.39	0.02	0.395
Self-reported family financial status				
Very poor	Ref			
Poor	− 0.08	0.53	− 0.01	0.885
Average	-1.97	0.42	− 0.18	< 0.001
Rich	-2.52	0.46	− 0.19	< 0.001
Very rich	-1.79	0.89	− 0.04	0.045

Note. B = unstandardized regression coefficient; SE = Standard error; β = standardized regression coefficient; Ref = Reference group. Missing data was excluded from data analysis

### Correlations Among Guilt/Shame Proneness (T1), MCER (T2), and Depressive Symptoms (T3)

Table 4 shown the correlations among the key variables. Relevant time points were selected to align with those used in the SEM (see Statistical Analysis). In summary, all but two correlations (between the GP-NBE at T1 and catastrophizing at T2; between the GP-RA at T1 and self-blame at T2) were of statistical significance. First, the two guilt proneness subscales at T1 were positively correlated with the two shame proneness subscales at T1 (*r* = .05 to 0.58; all *p* < .01). Interestingly, the two guilt proneness subscales at T1 were both negatively associated with depressive symptoms at T3 (*r* = − .09 to − 0.04; all *p* < .05) whereas the two shame proneness subscales at T1 were both positively associated with depressive symptoms at T3 (*r* = .15 to 0.20; all *p* < .001); the absolute sizes of the associations in the latter were stronger than those of the former. Third, the MCER variables (T2) were positively associated with depressive symptoms at T3 (*r* = .34 to 0.38; all *p* < .001). Fourth, the associations between the two shame proneness subscales and the MCER subscales (*r* = .11 to 0.21; all *p* < .001) were stronger than those between the two guilt proneness subscales and the MCER subscales (*r* = .05 to 0.07; *p* < .01).

**Table 4:** Correlation analysis

	1	2	3	4	5	6	7	8
1.GP-NBE T1	1							
2.GP-RA T1	0.64^***^	1						
3.SP-NSE T1	0.58^***^	0.45^***^	1					
4.SP-WA T1	0.10^***^	0.05^**^	0.34^***^	1				
5.Rumination T2	0.05^**^	0.05^**^	0.17^***^	0.13^***^	1			
6.Catastrophizing T2	−0 .01	−0 .05^**^	0.13^***^	0.19^***^	0.62^***^	1		
7.Self-blame T2	0.07^***^	0.03	0.21^***^	0.11^***^	0.53^***^	0.63^***^	1	
8.Depressive symptoms T3	− 0.04^*^	− 0.09^***^	0.15^***^	0.20^***^	0.34^***^	0.38^***^	0.36^***^	1

Note. T1 = baseline; T2 = 6-month follow-up; T3 = 12-month follow-up

*p < .05, **p <.01, ***p <.001

### SEM

**Fig. 1:**
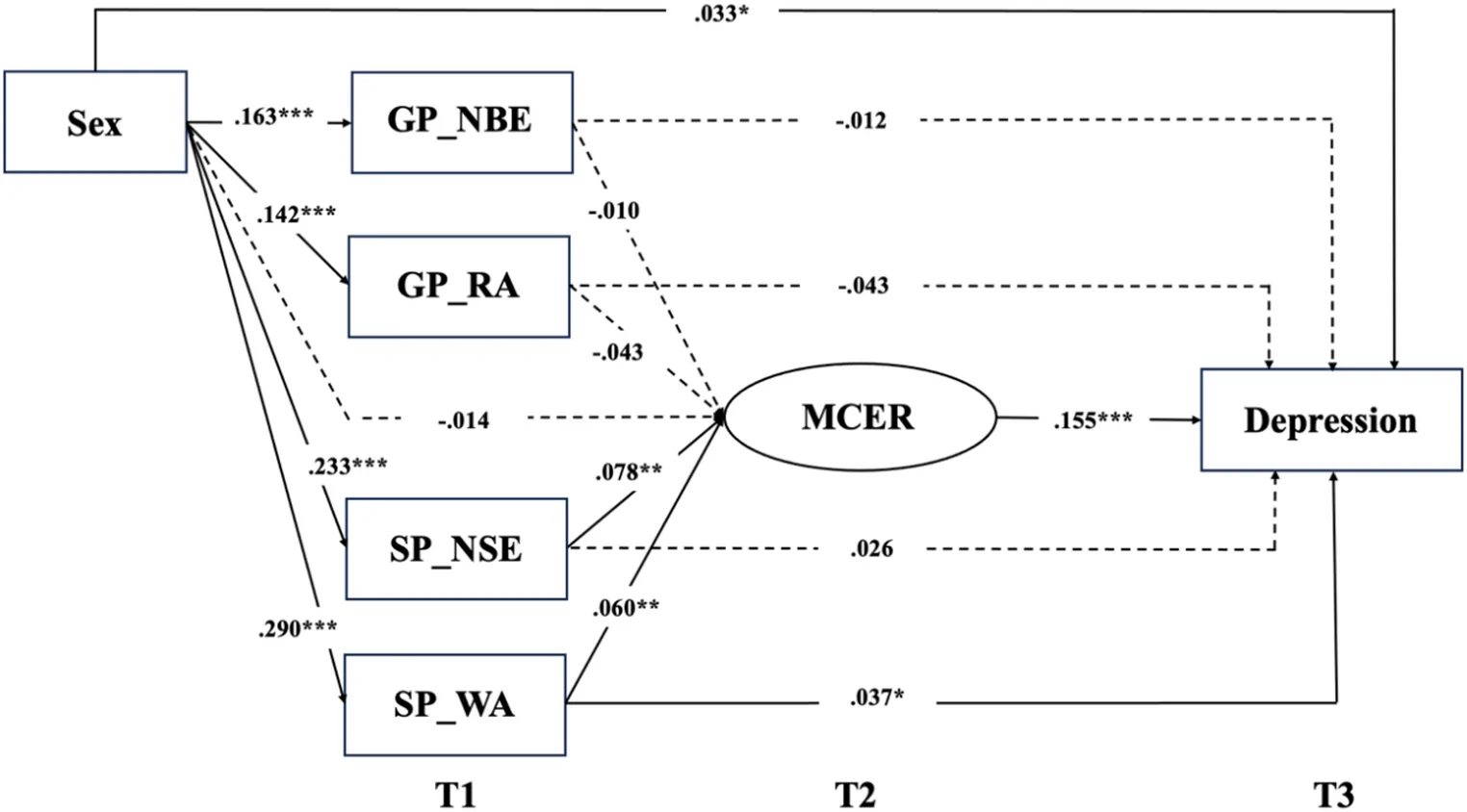
SEM of the mediation and suppression effects of shame/guilt proneness at T1 and MCER at T2 between sex and depressive symptoms at T3 (*N* = 2,915). The value of sex (1 = male, 2 = female). MCER was created as a second-order latent variable defined by three first-order factors (rumination, self-blame, and catastrophizing). Standardized coefficients were reported. The correlations of different variables at the same time are not presented in the model for simplification. *p < .05, **p <.01, ***p <.001

SEM showed satisfactory model fit indices (CFI = 0.921, RMSEA = 0.065, SRMR = 0.057). In Table 5; Figure 1, it is seen that three of the nine indirect paths (paths 4, 8, and 9) and the direct path between sex and depressive symptoms (T3) were statistically significant.

All three significant indirect paths involved the shame proneness subscales and the directions of such indirect paths were the same as that of the direct paths, they were mediation paths: (1) via the SP-WA subscale at T1, effect size 33.33%, *β* = 0.011, *p* = .036; (2) via SP-NSE at T1 and then MCER at T2 (a serial mediation path), effect size 9.09%, *β* = 0.003, *p* = .006; and (3) via SP-WA at T1 and MCER at T2 (a serial mediation), effect size 9.09%, *β* = 0.003, *p* = .006. In contrast, none of the four indirect paths involving the two guilt proneness subscales was statistically significant. Overall, Hypothesis 2 received partial support. Hypothesis 3 was supported only for shame, with the serial indirect effect through maladaptive cognitive emotion regulation emerging for shame but not for guilt. Similarly, Hypothesis 4 was supported only for shame, as the expected combination of mediation and suppression effects was observed for shame (mediation effect) but not for guilt (suppression effect). Here, the MCER was served as a second-order latent variable; detailed component-level results for each specific strategy were presented in the Appendix (Section SEM-Parallel Mediation).

**Table 5:** Mediation Analysis (*N* = 2,915)

Path	B	SE	95% CI	β	*p*	Effect size
**Total effect**	0.418	0.136	[0.152, 0.684]	0.044	**0.002**	-
**Direct effect**						
Sex → D T3	0.315	0.144	[0.032, 0.597]	0.033	**0.029**	-
**Indirect effect**	− 0.103	0.061	[-0.017, 0.223]	0.011	0.092	
Ind1: Sex → GP-NBE_T1 → D T3	− 0.018	0.039	[-0.094, 0.058]	− 0.002	0.646	6.06%
Ind2: Sex → GP-RA_T1 → D T3	− 0.059	0.033	[-0.123, 0.006]	− 0.006	0.073	18.18%
Ind3: Sex → SP-NSE_T1 → D T3	0.057	0.049	[-0.040, 0.153]	0.006	0.248	18.18%
Ind4: Sex SP_WA_T1D T3	0.103	0.049	[0.006, 0.199]	0.011	**0.036**	33.33%
Ind5: Sex → MCER_T2 → D T3	− 0.021	0.027	[-0.075, 0.033]	− 0.002	0.441	6.06%
Ind6: Sex → GP-NBE T1 → MCER _T2 → D T3	− 0.002	0.007	[-0.016, 0.011]	< 0.001	0.727	0.00%
Ind7: Sex → GP-RA T1 → MCER T2 → D T3	− 0.009	0.006	[-0.020, 0.002]	− 0.001	0.119	3.03%
Ind8: Sex → SP-NSE T1 → MCER T2 → D T3	0.027	0.010	[0.007, 0.046]	0.003	**0.006**	9.09%
Ind9: Sex → SP-WA T1 → MCER T2 → D T3	0.026	0.009	[0.007, 0.044]	0.003	**0.006**	9.09%

Note. D = Depressive symptoms, CI = Confidence interval. The models were adjusted for significant background characteristics (i.e., age, school type, father’s and mother’s educational level, family structure, living arrangement, self-reported family financial status), adjusted for the indirect influence of sex on MCER at T2 via MCER at T1, and adjusted for the indirect influence of sex on depressive symptoms at T3 via depressive symptoms at T2. Following the methodological recommendations for models with suppression effects (i.e., the symbol of the specific indirect effect is opposite to that of the direct effect) (Wen & Ye, 2014), the effect sizes of shame/guilt and MCER were calculated as the absolute ratio of the specific indirect effect to the direct effect, i.e., |ab/c’|×100%. The sum of individual indirect effect sizes may exceed 100% when suppression effects are present, as suppression and mediation effects operate in opposite directions

### Sensitivity Analysis

First, as the SP-WA subscale showed a low Cronbach alpha, a sensitivity SEM analysis was conducted by removing this subscale. This model showed satisfactory model fit indices, CFI = 0.920, RMSEA = 0.065, SRMR = 0.054. Moreover, the significance of the remaining scales and subscales were consistent with those shown in Table 5 (Ind8: Sex → SP-NSE T1 → MCER T2 → D T3). Detailed results were presented in Appendix (Section Sensitivity Analysis-SEM Excluding the Low-Reliability SP-WA Subscale).

Second, a complete case analysis with a subsample of 2,727 participants who had no missing values on all three waves was conducted as a sensitivity check. The current study re-conducted the SEM among the 2,727 complete cases, and the model fit stayed satisfactory, CFI = 0.904, RMSEA = 0.062, SRMR = 0.066. The statistical significance of all the proposed paths remained the same, except that: (1) the indirect path 2, i.e., Ind2: Sex → GP-RA T1 → D T3 became significant; (2) the indirect path 3, i.e., Ind2: Sex → SP-NSE T1 → D T3 became significant. Detailed results were presented in Appendix (Section Sensitivity Analysis-SEM with 2,727 Complete Cases).

In sum, the results of the two sensitivity analyses supported the robustness of the current findings.

## Discussion

Adolescence represents a critical developmental window frequently involving onsets of psychopathology (Kim-Cohen et al., 2003), along with formation and refinement of self and identity (Kroger, 2008). Sex disparity in mental distress emerges at young age and may adversely affect equity in life-long wellbeing (Bertha & Balázs, 2013; Johnson et al., 2009). The present study shed insights in understanding the sex disparity in adolescent depressive symptoms. Corroborating other studies (Else-Quest et al., 2012; Ma et al., 2021; Nolen-Hoeksema, 2012), the findings confirm sex disparities among females than among males – higher depressive symptoms, guilt proneness, shame proneness, and maladaptive cognitive emotional regulation. Whereas the two shame proneness subscales were positively associated with depressive symptoms, the two guilt proneness scales were negatively associated with depressive symptoms and with weaker absolute associations. In the SEM analysis, the observed gender disparity in depressive symptoms was mediated via the two shame proneness subscales (via SP-NSE and via SP-WA). However, the indirect paths between sex and depressive symptoms via guilt proneness were not significant. Maladaptive cognitive emotional regulation alone did not mediate between sex and depressive symptoms, but it mediated via serial mediations involving shame proneness but not guilt proneness. Most of these findings are novel and brings insight to understand the sex parity in depressive symptoms.

In this study, the prevalence of depressive symptoms (PHQ-9 ≥ 10) was approximately 28%, which aligns closely with recent meta-analytic estimates reported among Chinese adolescents (34.4%, Ma et al., 2021; 28.2%, Zhou et al., 2024) and falls within the range of global adolescent prevalence (14% − 64%; Shorey et al., 2022). Corroborating other studies (Reiss, 2013; Zajacova & Lawrence, 2018), socio-economic disadvantages (i.e., single-parenthood and living arrangement, low financial status and lower education level) were positively associated with depressive symptoms. The increasing age trend in prevalence of depressive symptoms, also found in other studies (Keyes et al., 2019; Thapar et al., 2012), should be considered when designing related intervention programs. The effect size of the in depressive symptoms was 0.27 (see Table 2) and the significant odds ratio for prevalence of depressive symptoms was 1.38 (see Table 3). While this effect is somewhat modest compared to the Cohen’s *d* of 0.41 reported for gender differences in depressive symptoms among adolescents (aged 16–19) across 90 nations (Salk et al., 2017), it is not negligible due to its significant implications.

The findings demonstrate robust gender divergence across affective (i.e., self-conscious emotions), cognitive (i.e., maladaptive cognitive emotion regulation), and psychopathological (i.e., depressive symptoms) domains. The elevated shame proneness and guilt proneness observed in females versus males align well with the relational orientation framework (Cross et al., 2011). As females often construct their identities through interpersonal connections, they are more susceptible to self-evaluative distress during the critical developmental window of identity consolidation (Rose & Rudolph, 2006). Furthermore, the data indicate that females utilize maladaptive cognitive emotional regulation strategies more frequently than males. This is consistent with gendered emotional regulation styles (Nolen-Hoeksema, 2012), which posit that females tend to employ internally focused, passive responses to distress, whereas males are more likely to engage in distraction or externalizing behaviors, such as substance use. Unlike the risk effects of shame proneness on depressive symptoms, this study showed that guilt proneness was negatively related with depressive symptoms and the absolute effect was milder than that of shame proneness, indicating a potentially protective effect against depressive symptoms. However, it should be noted that the correlations between guilt proneness and depressive symptoms, while statistically significant, were small in magnitude (*r* ≈ − .09 to − 0.04). These small effects might have reached significance due to the large sample size and should be interpreted with caution. Jointly, these findings underscore that female vulnerability for adolescent depressive symptoms is not due to a single deficit, but rather a multifaceted constellation of affective and cognitive burdens emerging in tandem. Therefore, efforts to close the sexual disparity must move beyond “symptom-specific” approaches; instead, a multi-pronged, gender-informed framework is required to holistically addresses the issue.

The findings provided an explanation for the sex difference in adolescent depressive symptoms. The sex gap was mainly mediated by shame proneness, i.e., female adolescents reported a higher level of shame proneness, which in turn predicted greater depressive symptomatology. Shame proneness is related to negative self-evaluation and withdrawal action tendency, which contribute to increase in depressive symptoms. Through targeting the global self, shame fostered pervasive self-devaluation that closely mirrored depressive schemas (Lewis, 2018). Shame-driven withdrawal exacerbated depressive outcomes, aligning with established associations between social withdrawal and depressive symptoms (Girard et al., 2014; Katz et al., 2011). However, although guilt proneness was negatively associated with depressive symptoms at the bivariate level, it did not emerge as a significant mediator in the pathway from sex to depressive outcomes. This pattern suggests that, while guilt is inversely related to depressive symptomatology, its role in explaining sex differences in depressive outcomes may be more limited than theoretically anticipated. Empirical research has repeatedly shown that shame is more robustly associated with depressive symptoms and maladaptive cognitive processes than guilt, which often exhibits null or weak effects once overlapping variance is controlled (Orth et al., 2006; Kim et al., 2011). In this context, the absence of a significant indirect effect in the present study may indicate that guilt does not function as a key intermediary mechanism linking sex differences to depressive outcomes in adolescent. Notably, the repair action dimension of guilt proneness was non-significant but showed a *p*-value of 0.073, which is worthy of attention and further confirmation.

The different effects of guilt and shame on depressive outcomes are interesting and implicative. The findings consolidate the claim supported by cross-sectional evidence that shame proneness and guilt proneness are closely but distinctive constructs leading to different motivational and cognitive consequences (Miceli & Castelfranchi, 2018; Orth et al., 2010). Shame involves a global devaluation of the self (e.g., “I am a bad person”) whereas guilt often focuses on specific behaviors such as “I did a bad thing” (Lewis, 2018). Thus, guilt may relate to adaptive behavioral correction rather than sustained cognitive-affective processing that drives depressive symptom development. While shame and guilt are intimately tied to self-construal across the lifespan (Lewis, 2018), adolescence represents a sensitive period where these emotions peak and diverge (Orth et al., 2010). Previous studies on the relationship between shame/guilt and depressive symptoms over-relied on adult cross-sectional samples (Muris & Meesters, 2014; Tangney & Tracy, 2012). This study partially filled out the knowledge gap.

Furthermore, the present study found that the indirect path from sex to depressive symptoms via maladaptive cognitive emotion regulation alone was non-significant (sex → maladaptive cognitive emotion regulation → depressive symptoms), whereas the serial pathway was significant exclusively through shame proneness (sex → shame proneness → maladaptive cognitive emotion regulation → depressive symptoms). The documented sex disparity in maladaptive cognitive emotion regulation may be a downstream consequence of heightened shame proneness (Oflazian & Borders, 2023; Orth et al., 2006). Shame, in particular, functioned as a potent dysregulating trigger that depleted the cognitive resources required for adaptive regulation (Lewis, 2018; Tangney et al., 2007). In the absence of this affective trigger, sex difference in the effect of maladaptive cognitive emotional regulation on depressive symptoms might diminish. In contrast, the serial pathway mediated by guilt was not significant, plausibly because guilt typically targets specific, controllable behaviors, preserving a sense of agency (Tangney & Dearing, 2003); the cognitive arousal associated with guilt is typically channeled into reparative planning or problem-solving (i.e., adaptive regulation) rather than devolving into the passive, perseverative cognitive styles characteristic of maladaptive cognitive emotion regulation (Miceli & Castelfranchi, 2018). Therefore, the hypothesized chain pathway (sex → guilt proneness→ maladaptive cognitive emotion regulation → depression) may not capture the primary functional role of guilt, which is more behaviorally oriented than cognitively mediational in nature.

Building upon these findings, interventions to reduce depressive symptoms should be multifaceted. To mitigate shame-proneness, health care workers may implement Compassion-Focused Therapy (CFT), which fosters “self-correction” rather than “self-condemnation” (Leaviss & Uttley, 2015). This approach facilitates a critical shift in the individual’s internal locus, moving from a globalized negative self-evaluation toward specific behavioral appraisals. Individuals can also enhance metacognitive awareness to attenuate maladaptive cognitive emotion regulation. By fostering real-time monitoring of regulatory cycles, individuals can interrupt the habitual rumination and catastrophizing that characterize depressive entrenchment (Pennequin et al., 2019). Collectively, these strategies function by extinguishing maladaptive affective triggers (shame), reframing shame-based self-evaluations into more specific behavior-focused appraisals, and disrupting the rigid regulatory routines (maladaptive cognitive emotion regulation), thereby providing a robust psychological buffer against depressive symptoms.

This study has several limitations. First, the Cronbach alpha of SP-WA was low; it warrants cautious interpretation of related findings. Similar low reliability for the SP-WA subscale has been reported in other validation studies. For instance, recent Western validations report an alpha of 0.60 for this subscale (Veneziani et al., 2024). This pattern is equally evident in Chinese adaptations, where alphas generally range from 0.45 to 0.58 (Tian, 2024; Wang et al., 2016). This reduced internal consistency is largely attributable to the scenario-based format of the measure, in which each item captures not only the underlying psychological construct but also the context-specific variables embedded within the respondent’s reaction to distinct situations, inherently introducing situational variability across items (Cohen et al., 2011). Future research should replicate these pathways using other scales assessing guilt and shame proneness and/or to validate GASP in various cultures. Second, as those with depressive symptoms and higher levels of guilt/shame proneness might be less willing to present socially undesirable responses, reporting bias might exist. Third, the sample was confined to students of a Chinese city and generalizations to other parts of China and countries need caution, keeping in mind that shame and guilt reflect violation of social norms (Tangney et al., 2007; Tangney & Tracy, 2012), which are culturally specific (Cross et al., 2011; Gelfand et al., 2011). Fourth, the study only looked as three mediation/suppression variables, other variables and mechanisms (e.g., resilience, social support, emotional expression) have not been investigated. Fifth, although the present study adopts a longitudinal design with repeated measurements of guilt, shame, maladaptive cognitive emotion regulation, and depressive symptoms across three waves, it is important to acknowledge that the analytic strategy was intentionally specified as a theory-driven, time-ordered mediation model rather than a fully dynamic reciprocal system. Future research employing cross-lagged panel models or random-intercept cross-lagged panel models (RI-CLPM) may further extend the present findings by examining reciprocal developmental processes among guilt/shame, maladaptive cognitive emotion regulation, and depressive symptoms over time. Such approaches would allow for a more comprehensive assessment of bidirectional and dynamic associations among these constructs.

## Conclusion

Despite well-documented sex differences in adolescent depressive symptoms, the specific emotional mechanisms underlying this disparity have received limited longitudinal investigation. To address this gap, the current three-wave longitudinal study examined the differential mediating roles of guilt proneness and shame proneness via maladaptive cognitive emotion regulation strategies to specifically illuminate this sex disparity. The results revealed that shame proneness mediated the sex disparity in depressive symptoms, both directly and serially through maladaptive cognitive emotion regulation strategies, whereas guilt proneness did not function as a mediator. These findings suggest that the sex disparity in adolescent depressive symptoms is partially maintained by elevated shame proneness among female adolescents, which in turn promotes maladaptive cognitive emotion regulation patterns that heighten depressive risk. This study advances the understanding of adolescent development by illuminating how sex-differentiated self-conscious emotions distinctly shape cognitive coping trajectories to drive depressive symptoms. Ultimately, interventions that reduce shame-based self-evaluations, reframe them into behavior-focused appraisals, and disrupt maladaptive regulatory routines may provide a robust buffer against depressive symptoms during this critical developmental stage.

## Supplementary Information

Below is the link to the electronic supplementary material.

Supplementary Material 1

## Data Availability

The datasets generated and/or analyzed during the current study are not publicly available but are available from the corresponding author on reasonable request.

## Footnotes

1 The four subscales were retained as separate indicators rather than aggregated into composite scores for both conceptual and empirical reasons. Conceptually, the GASP distinguishes between two theoretically distinct components of both guilt and shame (Cohen et al., 2011). Empirically, these subscales have been shown to exhibit differential associations with psychopathology and behavior (Cohen et al., 2011; Tian, 2024; Wang et al., 2016; Xiao et al., 2023). Aggregating them would lose potentially meaningful variance and obscure the specific mechanisms through which guilt and shame relate to depressive symptoms. This is also consistent with the original four-factor, rather than three-factor or two-factor, structure of the GASP.

2 Measurement invariance test of the MCER construct was examined across sex and across the three time points to ensure that observed sex differences and longitudinal associations in MCER were not attributable to measurement artifacts. Multi-group confirmatory factor analyses supported configural, metric, and scalar invariance across males and females at each wave, as well as longitudinal invariance across T1–T3. Changes in model fit indices were within recommended thresholds (ΔCFI ≤ 0.010, ΔRMSEA ≤ 0.015), indicating that MCER was measured equivalently across sex and time. Therefore, subsequent comparisons of sex differences and longitudinal associations were considered appropriate. Detailed invariance testing results are presented in Appendix (Section Measurement Invariance).

## References

[CR1] Bertha, E. A., & Balázs, J. (2013). Subthreshold depression in adolescence: A systematic review. *European Child & Adolescent Psychiatry*, *22*(10), 589–603. 10.1007/s00787-013-0411-0 23579389

[CR71] Chengli Huang is a Lecturer at the Wenzhou Medical University Her major research interests include adolescent mental health.

[CR2] Cheung, M. P., Gilbert, P., & Irons, C. (2004). An exploration of shame, social rank and rumination in relation to depression. *Personality and Individual Differences*, *36*(5), 1143–1153. 10.1016/S0191-8869(03)00206-X

[CR3] Cohen, T. R., Wolf, S. T., Panter, A. T., & Insko, C. A. (2011). Introducing the GASP scale: A new measure of guilt and shame proneness. *Journal of Personality and Social Psychology*, *100*(5), 947–966. 10.1037/a0022641 21517196

[CR4] Collaborators. (2022). Global, regional, and national burden of 12 mental disorders in 204 countries and territories, 1990–2019: A systematic analysis for the Global Burden of Disease Study 2019. *The Lancet Psychiatry*, *9*(2), 137–150. 10.1016/S2215-0366(21)00395-3 PMC8776563 35026139

[CR5] Cross, S. E., Hardin, E. E., & Gercek-Swing, B. (2011). The what, how, why, and where of self-construal. *Personality and Social Psychology Review*, *15*(2), 142–179. 10.1177/1088868310373752 20716643

[CR6] Cyranowski, J. M., Frank, E., Young, E., & Shear, M. K. (2000). Adolescent onset of the gender difference in lifetime rates of major depression: A theoretical model. *Archives of General Psychiatry*, *57*(1), 21–27. 10.1001/archpsyc.57.1.21 10632229

[CR7] Else-Quest, N. M., Higgins, A., Allison, C., & Morton, L. C. (2012). Gender differences in self-conscious emotional experience: A meta-analysis. *Psychological Bulletin*, *138*(5), 947–981. 10.1037/a0027930 22468881

[CR8] Estévez, A., Momeñe, J., Jauregui, P., & Etxaburu, N. (2024). Shame and blame in gambling: Relationship with emotion regulation and gambling motives. *International Journal of Mental Health and Addiction*, *22*(4), 2012–2027. 10.1007/s11469-022-00970-3

[CR9] Ferrari, A. J., Charlson, F. J., Norman, R. E., Patten, S. B., Freedman, G., Murray, C. J. L., Vos, T., & Whiteford, H. A. (2013). Burden of depressive disorders by country, sex, age, and year: Findings from the global burden of disease study 2010. *PLoS Medicine*, *10*(11), e1001547. 10.1371/journal.pmed.1001547 PMC3818162 24223526

[CR10] Garnefski, N., & Kraaij, V. (2006). Cognitive emotion regulation questionnaire–development of a short 18-item version (CERQ-short). *Personality and Individual Differences*, *41*(6), 1045–1053. 10.1016/j.paid.2006.04.010

[CR11] Gavazzi, I. G., Ornaghi, V., & Antoniotti, C. (2011). Children’s and adolescents’ narratives of guilt: Antecedents and mentalization. *European Journal of Developmental Psychology*, *8*(3), 311–330. 10.1080/17405629.2010.491303

[CR12] Gelfand, M. J., Raver, J. L., Nishii, L., Leslie, L. M., Lun, J., Lim, B. C., Duan, L., Almaliach, A., Ang, S., Arnadottir, J., Aycan, Z., Boehnke, K., Boski, P., Cabecinhas, R., Chan, D., Chhokar, J., D’Amato, A., Subirats Ferrer, M., Fischlmayr, I. C., & Yamaguchi, S. (2011). Differences between tight and loose cultures: A 33-nation study. *Science*, *332*(6033), 1100–1104. 10.1126/science.1197754 21617077

[CR13] Girard, J. M., Cohn, J. F., Mahoor, M. H., Mavadati, S. M., Hammal, Z., & Rosenwald, D. P. (2014). Nonverbal social withdrawal in depression: Evidence from manual and automatic analyses. *Image and Vision Computing*, *32*(10), 641–647. 10.1016/j.imavis.2013.12.007 PMC4217695 25378765

[CR14] Girgus, J. S., & Yang, K. (2015). Gender and depression. *Current Opinion in Psychology Depression*, *4*, 53–60. 10.1016/j.copsyc.2015.01.019

[CR15] Hyde, J. S., & Mezulis, A. H. (2020). Gender differences in depression: Biological, affective, cognitive, and sociocultural factors. *Harvard Review of Psychiatry*, *28*(1), 4–13. 10.1097/HRP.0000000000000230 31913978

[CR16] Hyde, J. S., Mezulis, A. H., & Abramson, L. Y. (2008). The ABCs of depression: Integrating affective, biological, and cognitive models to explain the emergence of the gender difference in depression. *Psychological Review*, *115*(2), 291–313. 10.1037/0033-295X.115.2.291 18426291

[CR17] Johnson, D. P., & Whisman, M. A. (2013). Gender differences in rumination: A meta-analysis. *Personality and Individual Differences*, *55*(4), 367–374. 10.1016/j.paid.2013.03.019 PMC3786159 24089583

[CR18] Johnson, J. G., Cohen, P., & Kasen, S. (2009). Minor depression during adolescence and mental health outcomes during adulthood. *British Journal of Psychiatry*, *195*(3), 264–265. 10.1192/bjp.bp.108.054239 PMC2801821 19721119

[CR19] Katz, S. J., Conway, C. C., Hammen, C. L., Brennan, P. A., & Najman, J. M. (2011). Childhood social withdrawal, interpersonal impairment, and young adult depression: A mediational model. *Journal of Abnormal Child Psychology*, *39*(8), 1227–1238. 10.1007/s10802-011-9537-z PMC3384496 21744059

[CR21] Keyes, K. M., & Platt, J. M. (2024). Annual Research Review: Sex, gender, and internalizing conditions among adolescents in the 21st century – trends, causes, consequences. *Journal of Child Psychology and Psychiatry*, *65*(4), 384–407. 10.1111/jcpp.13864 PMC12341061 37458091

[CR20] Keyes, K. M., Gary, D., O’Malley, P. M., Hamilton, A., & Schulenberg, J. (2019). Recent increases in depressive symptoms among US adolescents: Trends from 1991 to 2018. *Social Psychiatry and Psychiatric Epidemiology*, *54*(8), 987–996. 10.1007/s00127-019-01697-8 PMC7015269 30929042

[CR22] Kim, S., Thibodeau, R., & Jorgensen, R. S. (2011). Shame, guilt, and depressive symptoms: A meta-analytic review. *Psychological Bulletin*, *137*(1), 68–96. 10.1037/a0021466 21219057

[CR23] Kim-Cohen, J., Caspi, A., Moffitt, T. E., Harrington, H., Milne, B. J., & Poulton, R. (2003). Prior juvenile diagnoses in adults with mental disorder: Developmental follow-back of a prospective-longitudinal cohort. *Archives of General Psychiatry*, *60*(7), 709–717. 10.1001/archpsyc.60.7.709 12860775

[CR24] Kline, R. B. (2023). *Principles and practice of structural equation modeling*. Guilford.

[CR25] Kochanska, G., Gross, J. N., Lin, M. H., & Nichols, K. E. (2002). Guilt in young children: Development, determinants, and relations with a broader system of standards. *Child Development*, *73*(2), 461–482. 10.1111/jcpp.13864 11949903

[CR26] Kroenke, K., Spitzer, R. L., & Williams, J. B. W. (2001). The PHQ-9: Validity of a brief depression severity measure. *Journal of General Internal Medicine*, *16*(9), 606–613. 10.1046/j.1525-1497.2001.016009606.x PMC1495268 11556941

[CR27] Kroger, J. (2008). Identity development during adolescence. In G. R. Adams & M. D. Berzonsky (Eds.), *Blackwell handbook of adolescence* (pp. 205–226). Blackwell Pub.

[CR28] Leaviss, J., & Uttley, L. (2015). Psychotherapeutic benefits of compassion-focused therapy: An early systematic review. *Psychological Medicine*, *45*(5), 927–945. 10.1017/S0033291714002141 PMC4413786 25215860

[CR29] Leung, D. Y. P., Mak, Y. W., Leung, S. F., Chiang, V. C. L., & Loke, A. Y. (2020). Measurement invariances of the PHQ-9 across gender and age groups in Chinese adolescents. *Asia-Pacific Psychiatry*, *12*(3), e12381. 10.1111/appy.12381 PMC7507123 32011101

[CR30] Lewis, M. (2018). Self-conscious emotions: Embarrassment, pride, shame, guilt, and hubris. In *Handbook of emotions* (Fourth Edition, pp. 792–814). The Guilford Press.

[CR31] Liw, L., Ciftci, A., & Kim, T. (2022). Cultural values, shame and guilt, and expressive suppression as predictors of depression. *International Journal of Intercultural Relations*, *89*, 90–99. 10.1016/j.ijintrel.2022.05.005

[CR32] Ma, L., Mazidi, M., Li, K., Li, Y., Chen, S., Kirwan, R., Zhou, H., Yan, N., Rahman, A., & Wang, W. (2021). Prevalence of mental health problems among children and adolescents during the COVID-19 pandemic: A systematic review and meta-analysis. *Journal of Affective Disorders*, *293*, 78–89. 10.1016/j.jad.2021.06.021 PMC9711885 34174475

[CR33] Miceli, M., & Castelfranchi, C. (2018). Reconsidering the differences between shame and guilt. *Europe’s Journal of Psychology*, *14*(3), 710–733. 10.5964/ejop.v14i3.1564 PMC6143989 30263080

[CR34] Muris, P., & Meesters, C. (2014). Small or big in the eyes of the other: On the developmental psychopathology of self-conscious emotions as shame, guilt, and pride. *Clinical Child and Family Psychology Review*, *17*(1), 19–40. 10.1007/s10567-013-0137-z 23712881

[CR35] Needham, B. L. (2009). Adolescent depressive symptomatology and young adult educational attainment: An examination of gender differences. *Journal of Adolescent Health*, *45*(2), 179–186. 10.1016/j.jadohealth.2008.12.015 19628145

[CR36] Nolen-Hoeksema, S. (2012). Emotion regulation and psychopathology: The role of gender. *Annual Review of Clinical Psychology*, *8*, 161–187. 10.1146/annurev-clinpsy-032511-143109 22035243

[CR37] Nolen-Hoeksema, S., Wisco, B. E., & Lyubomirsky, S. (2008). Rethinking rumination. *Perspectives on Psychological Science*, *3*(5), 400–424. 10.1111/j.1745-6924.2008.00088.x 26158958

[CR38] Oflazian, J. S., & Borders, A. (2023). Does rumination mediate the unique effects of shame and guilt on procrastination? *Journal of Rational-Emotive and Cognitive-Behavior Therapy*, *41*(1), 237–246. 10.1007/s10942-022-00466-y PMC9274181 35847054

[CR39] Orth, U., Berking, M., & Burkhardt, S. (2006). Self-conscious emotions and depression: Rumination explains why shame but not guilt is maladaptive. *Personality and Social Psychology Bulletin*, *32*(12), 1608–1619. 10.1177/0146167206292958 17122174

[CR40] Orth, U., Robins, R. W., & Soto, C. J. (2010). Tracking the trajectory of shame, guilt, and pride across the life span. *Journal of Personality and Social Psychology*, *99*(6), 1061–1071. 10.1037/a0021342 21114354

[CR41] Parker, G., & Brotchie, H. (2010). Gender differences in depression. *International Review of Psychiatry*, *22*(5), 429–436. 10.3109/09540261.2010.492391 21047157

[CR42] Pennequin, V., Questel, F., Delaville, E., Delugre, M., & Maintenant, C. (2019). Metacognition and emotional regulation in children from 8 to 12 years old. *British Journal of Educational Psychology*, *90*, 1–16. 10.1111/bjep.12305 31292954

[CR43] Reiss, F. (2013). Socioeconomic inequalities and mental health problems in children and adolescents: A systematic review. *Social Science & Medicine*, *90*, 24–31. 10.1016/j.socscimed.2013.04.026 23746605

[CR44] Rose, A. J., & Rudolph, K. D. (2006). A review of sex differences in peer relationship processes: Potential trade-offs for the emotional and behavioral development of girls and boys. *Psychological Bulletin*, *132*(1), 98–131. 10.1037/0033-2909.132.1.98 PMC3160171 16435959

[CR45] Salk, R. H., Hyde, J. S., & Abramson, L. Y. (2017). Gender differences in depression in representative national samples: Meta-analyses of diagnoses and symptoms. *Psychological Bulletin*, *143*(8), 783–822. 10.1037/bul0000102 PMC5532074 28447828

[CR46] Schafer, J. L., & Graham, J. W. (2002). Missing data: Our view of the state of the art. *Psychological Methods*, *7*(2), 147–177. 10.1037/1082-989X.7.2.147 12090408

[CR47] Sebastian, C., Burnett, S., & Blakemore, S. J. (2008). Development of the self-concept during adolescence. *Trends in Cognitive Sciences*, *12*(11), 441–446. 10.1016/j.tics.2008.07.008 18805040

[CR48] Shao, H., Du, M., Wang, D. B., Wu, X., Ren, Z., Yu, Y., Wu, A. M. S., Zhang, G., Lu, H., Xu, D., Wu, J., Tao, L., Zhuge, X., & Lau, J. T. F. (2026). Sex differences in social anxiety: A longitudinal serial mediation via perceived stress and resilience among adolescents in China. *Journal of Affective Disorders*, *392*, 120212. 10.1016/j.jad.2025.120212 40912330

[CR49] Shore, L., Toumbourou, J. W., Lewis, A. J., & Kremer, P. (2018). Longitudinal trajectories of child and adolescent depressive symptoms and their predictors – a systematic review and meta-analysis. *Child and Adolescent Mental Health*, *23*(2), 107–120. 10.1111/camh.12220 32677332

[CR50] Shorey, S., Ng, E. D., & Wong, C. H. J. (2022). Global prevalence of depression and elevated depressive symptoms among adolescents: A systematic review and meta-analysis. *British Journal of Clinical Psychology*, *61*(2), 287–305. 10.1111/bjc.12333 34569066

[CR51] Szentágotai-Tătar, A., & Miu, A. C. (2016). Individual differences in emotion regulation, childhood trauma and proneness to shame and guilt in adolescence. *PLOS ONE*, *11*(11), e0167299. 10.1371/journal.pone.0167299 PMC5127568 27898709

[CR52] Sznycer, D. (2019). Forms and functions of the self-conscious emotions. *Trends in Cognitive Sciences*, *23*(2), 143–157. 10.1016/j.tics.2018.11.007 30583948

[CR53] Tangney, J. P., & Dearing, R. L. (2003). *Shame and guilt*. Guilford Press.

[CR55] Tangney, J. P., & Tracy, J. L. (2012). Self-conscious emotions. In M. R. Leary & J. P. Tangney (Eds.), *Handbook of self and identity* (pp. 446–478). Guilford Press. (The Second Editor.

[CR54] Tangney, J. P., Stuewig, J., & Mashek, D. J. (2007). Moral emotions and moral behavior. *Annual Review of Psychology*, *58*(1), 345–372. 10.1146/annurev.psych.56.091103.070145 PMC3083636 16953797

[CR56] Thapar, A., Collishaw, S., Pine, D. S., & Thapar, A. K. (2012). Depression in adolescence. *The Lancet*, *379*(9820), 1056–1067. 10.1016/S0140-6736(11)60871-4 PMC3488279 22305766

[CR57] Thapar, A., Eyre, O., Patel, V., & Brent, D. (2022). Depression in young people. *The Lancet*, *400*(10352), 617–631. 10.1016/S0140-6736(22)01012-1 35940184

[CR58] Tian, Y. (2024). *The relationship between narcissism and peer status in junior high school students: The mediating role of self-conscious emotions [Master’s Thesis]*. Ludong University.

[CR59] Tilghman-Osborne, C., Cole, D. A., Felton, J. W., & Ciesla, J. A. (2008). Relation of guilt, shame, behavioral and characterological self-blame to depressive symptoms in adolescents over time. *Journal of Social and Clinical Psychology*, *27*(8), 809–842. 10.1521/jscp.2008.27.8.809 PMC4238306 25419043

[CR60] Veneziani, G., Ciacchella, C., Onorati, P., & Lai, C. (2024). Attachment theory 2.0: A network analysis of offline and online attachment dimensions, guilt, shame, and self-esteem and their differences between low and high internet users. *Computers in Human Behavior*, *156*, 108195. 10.1016/j.chb.2024.108195

[CR62] Wang, X., Zhan, Y., & Yan, L. (2016). Reliability and validity test of Chinese version of the guilt and shame proneness scale. *Chinese Journal of Clinical Psychology*, *24*(5), 865–868.

[CR61] Wang, C., Tong, Y., Tang, T., Wang, X., Fang, L., Wen, X., Su, P., Wang, J., & Wang, G. (2024). Association between adolescent depression and adult suicidal behavior: A systematic review and meta-analysis. *Asian Journal of Psychiatry*, *100*, 104185. 10.1016/j.ajp.2024.104185 39106588

[CR63] Weavers, B., Heron, J., Thapar, A. K., Stephens, A., Lennon, J., Bevan Jones, R., Eyre, O., Anney, R. J., Collishaw, S., Thapar, A., & Rice, F. (2021). The antecedents and outcomes of persistent and remitting adolescent depressive symptom trajectories: A longitudinal, population-based English study. *The Lancet Psychiatry*, *8*(12), 1053–1061. 10.1016/S2215-0366(21)00281-9 34672991

[CR64] Webb, M., Heisler, D., Call, S., Chickering, S. A., & Colburn, T. A. (2007). Shame, guilt, symptoms of depression, and reported history of psychological maltreatment. *Child Abuse & Neglect*, *31*(11), 1143–1153. 10.1016/j.chiabu.2007.09.003 18023873

[CR65] Wen, Z., & Ye, B. (2014). Analyses of mediating effects: The development of methods and models. *Advances in Psychological Science*, *22*(5), 731–745. 10.3724/SP.J.1042.2014.00731

[CR66] Xiao, F., Zhao, J., Fan, L., Ji, X., Fang, S., Zhang, P., Kong, X., Liu, Q., Yu, H., Zhou, X., Gao, X., & Wang, X. (2023). Understanding guilt-related interpersonal dysfunction in obsessive-compulsive personality disorder through computational modeling of two social interaction tasks. *Psychological Medicine*, *53*(12), 5569–5581. 10.1017/S003329172200277X 36066201

[CR67] Yu, Y., Ng, J. H. Y., Wang, Z., Tian, X., & Lau, J. T. F. (2024). University students who were men who had sex with men (MSM) in Sichuan, China had a higher prevalence of insomnia and probable depression than their non-MSM counterparts: Mediation via emotional dysregulations. *Bmc Psychiatry*, *24*, 793. 10.1186/s12888-024-06192-2 PMC11559193 39533253

[CR68] Zajacova, A., & Lawrence, E. M. (2018). The relationship between education and health: Reducing disparities through a contextual approach. *Annual Review of Public Health*, *39*(1), 273–289. 10.1146/annurev-publhealth-031816-044628 PMC5880718 29328865

[CR69] Zhou, J., Liu, Y., Ma, J., Feng, Z., Hu, J., Hu, J., & Dong, B. (2024). Prevalence of depressive symptoms among children and adolescents in China: A systematic review and meta-analysis. *Child and Adolescent Psychiatry and Mental Health*, *18*, 150. 10.1186/s13034-024-00841-w PMC11577650 39563377

