# Supplementary material for: Guilt Proneness and Shame Proneness Differentially Relate to Sex Disparity in Depression Among Adolescents in China: A Longitudinal Serial Mediation Analysis
Source: J Youth Adolesc. 2026 Aug 26;55(10):2634–47. doi: 10.1007/s10964-026-02403-z (PMC13695586; doi:10.1007/s10964-026-02403-z)
Supplement: Supplementary file 1 [file 10964_2026_2403_MOESM1_ESM.docx]

**Guilt Proneness and Shame Proneness Differentially Relate to Sex Disparity in Depression Among Adolescents in China: A Longitudinal Serial Mediation Analysis**

**Attrition Analyses**

Completers: n = 2,915; Dropouts: n = 819.

Continuous variables were compared using Welch's t-test. Categorical variables were compared using Pearson's chi-square test. Effect sizes are reported as Cohen's d for continuous variables and Cramer's V for categorical variables.

Table S1. Descriptive Statistics and Comparisons for Continuous Variables by Group

| Variable | Completer | | | Dropout | | | *t* | *p* | Cohen’s *d* |
| --- | --- | --- | --- | --- | --- | --- | --- | --- | --- |
|  | N | Mean | SD | N | Mean | SD |  |  |  |
| Age | 2,915 | 17.00 | 1.33 | 817 | 17.42 | 1.36 | -7.83 | **< .001** | -0.31 |
| Depressive symptoms | 2,907 | 7.47 | 5.02 | 816 | 8.02 | 5.67 | -2.50 | **0.013** | -0.10 |
| Rumination | 2,899 | 5.51 | 2.11 | 812 | 5.58 | 2.21 | -0.79 | 0.433 | -0.03 |
| Catastrophizing | 2,898 | 4.10 | 2.16 | 811 | 4.33 | 2.27 | -2.48 | **0.013** | -0.10 |
| Self-blame | 2,898 | 4.48 | 2.00 | 811 | 4.53 | 2.12 | -0.53 | 0.595 | -0.02 |
| GP-NBE | 2,908 | 5.65 | 1.13 | 817 | 5.55 | 1.21 | 2.09 | **0.037** | 0.08 |
| GP-RA | 2,910 | 5.45 | 1.08 | 817 | 5.31 | 1.15 | 3.08 | **0.002** | 0.12 |
| SP-NSE | 2,909 | 4.87 | 1.24 | 817 | 4.79 | 1.30 | 1.61 | 0.109 | 0.06 |
| SP-WA | 2,909 | 3.73 | 1.13 | 817 | 3.75 | 1.19 | -0.36 | 0.717 | -0.02 |

Note. SD = Standard deviation. GP-NBE = Guilt Proneness-Negative Behavior Evaluation subscale; GP-RA = Guilt Proneness-Repair Action Subscale; SP-NSE = Shame Proneness-Negative Self Evaluation subscale; SP-WA = Shame Proneness-Repair Action Subscale.

Table S2. Group Comparisons for Categorical Variables

| Variable | *χ^2^* | *df* | *p* | Cramer’s *V* |
| --- | --- | --- | --- | --- |
| School type | 37.15 | 2 | **< .001** | 0.10 |
| Sex | 1.69 | 1 | 0.193 | 0.02 |
| Single parenthood | 7.75 | 1 | **0.005** | 0.05 |
| Living arrangement | 2.44 | 3 | 0.487 | 0.03 |
| Father’s education level | 1.31 | 4 | 0.861 | 0.02 |
| Mother’s education level | 7.12 | 4 | 0.130 | 0.05 |
| Self-reported family financial status | 6.35 | 5 | 0.274 | 0.04 |

Table S3.1 Descriptive Statistics for School Type

| Level | Completer | Dropout | Completer percentage | Dropout percentage |
| --- | --- | --- | --- | --- |
| Junior middle school | 692 | 114 | 23.7 | 13.9 |
| Senior high school | 807 | 268 | 27.7 | 32.7 |
| Vocational high school | 1,416 | 437 | 48.6 | 53.4 |

Table S3.2 Descriptive Statistics for Sex

| Level | Completer | Dropout | Completer percentage | Dropout percentage |
| --- | --- | --- | --- | --- |
| Male | 1,747 | 466 | 59.9 | 57.3 |
| Female | 1,168 | 347 | 40.1 | 42.7 |

Table S3.3 Descriptive Statistics for Single Parenthood

| Level | Completer | Dropout | Completer percentage | Dropout percentage |
| --- | --- | --- | --- | --- |
| No | 2,474 | 590 | 84.9 | 72.0 |
| Yes | 274 | 94 | 9.4 | 11.5 |
| Not reported | 167 | 53 | 5.7 | 6.5 |

Table S3.4 Descriptive Statistics for Living Arrangement

| Level | Completer | Dropout | Completer percentage | Dropout percentage |
| --- | --- | --- | --- | --- |
| Co-residing with both parents | 2,194 | 594 | 75.3 | 72.8 |
| Co-residing with mother | 302 | 89 | 10.4 | 10.9 |
| Co-residing with father | 141 | 47 | 4.8 | 5.8 |
| Co-residing with neither | 278 | 86 | 9.5 | 10.5 |

Table S3.5 Descriptive Statistics for Father’s Education Level

| Level | Completer | Dropout | Completer percentage | Dropout percentage |
| --- | --- | --- | --- | --- |
| Elementary or lower | 338 | 98 | 12.0 | 12.7 |
| Middle school | 1,312 | 372 | 46.5 | 48.1 |
| High school | 804 | 210 | 28.5 | 27.1 |
| Post-secondary | 155 | 40 | 5.5 | 5.2 |
| College or higher | 212 | 54 | 7.5 | 7.0 |
| Elementary or lower | 338 | 98 | 12.0 | 12.7 |

Table S3.6 Descriptive Statistics for Mother’s Education Level

| Level | Completer | Dropout | Completer percentage | Dropout percentage |
| --- | --- | --- | --- | --- |
| Elementary or lower | 498 | 140 | 17.7 | 18.2 |
| Middle school | 1,265 | 341 | 44.9 | 44.2 |
| High school | 665 | 199 | 23.6 | 25.8 |
| Post-secondary | 165 | 49 | 5.9 | 6.4 |
| College or higher | 227 | 42 | 8.0 | 5.4 |
| Elementary or lower | 498 | 140 | 17.7 | 18.2 |
| Middle school | 1,265 | 341 | 44.9 | 44.2 |

Table S3.7 Descriptive Statistics for Self-reported Family Financial Status

| Level | Completer | Dropout | Completer percentage | Dropout percentage |
| --- | --- | --- | --- | --- |
| Very poor | 47 | 21 | 1.6 | 2.6 |
| Poor | 203 | 70 | 7.0 | 8.6 |
| Average | 2,033 | 552 | 69.7 | 67.7 |
| Rich | 487 | 129 | 16.7 | 15.8 |
| Very rich | 39 | 12 | 1.3 | 1.5 |
| Not clear | 106 | 31 | 3.6 | 3.8 |

**Measurement Invariance**

**Methods**

To ensure that observed sex differences and longitudinal associations were not attributable to measurement artifacts, measurement invariance of the maladaptive cognitive emotion regulation (MCER) construct was examined across sex and across time using multi-group confirmatory factor analysis in Mplus 8.3. For sex invariance, separate analyses were conducted at T1, T2, and T3, comparing males and females. For longitudinal invariance, MCER was examined simultaneously across the three waves (T1–T3). Following standard procedures, configural, metric, and scalar invariance models were tested sequentially (Vandenberg & Lance, 2000). Configural invariance evaluated whether the same factor structure held across groups or time points. Metric invariance additionally constrained factor loadings to equality, whereas scalar invariance further constrained item intercepts to equality. Model comparisons were evaluated using changes in comparative fit index (ΔCFI) and root mean square error of approximation (ΔRMSEA). Following recommended guidelines, invariance was supported when ΔCFI ≤ .010 and ΔRMSEA ≤ .015 (Chen, 2007; Cheung & Rensvold, 2002).

**Results**

As shown in Tables S4 and S5, configural, metric, and scalar invariance were supported across males and females at all three waves. Changes in model fit were minimal (ΔCFI = .001–.005; ΔRMSEA = .000–.009), remaining well below recommended thresholds. Similarly, longitudinal measurement invariance analyses across T1–T3 supported configural, metric, and scalar invariance. Constraining factor loadings and item intercepts across time resulted in only trivial decreases in model fit (ΔCFI ≤ .004; ΔRMSEA ≤ .002). These findings indicate that MCER was measured equivalently across sex and across time, supporting the interpretation of sex differences and longitudinal relationships reported in the present study.

**Table S4** Measurement Invariance of MCER Across Sex.

| Model | *χ^2^* | *df* | CFI | RMSEA [90% CI] | ∆CFI | ∆RMSEA |
| --- | --- | --- | --- | --- | --- | --- |
| Time 1 |  |  |  |  |  |  |
| M0 (configural) | 100.03 | 12 | 0.985 | 0.071 [0.059, 0.084] | — | — |
| M1 (metric) | 112.91 | 17 | 0.984 | 0.062 [0.052, 0.074] | 0.001 | 0.009 |
| M2 (scalar) | 124.65 | 22 | 0.982 | 0.057 [0.047, 0.067] | 0.002 | 0.005 |
| Time 2 |  |  |  |  |  |  |
| M0 (configural) | 92.10 | 12 | 0.987 | 0.069 [0.056, 0.082] | — | — |
| M1 (metric) | 114.89 | 17 | 0.985 | 0.063 [0.052, 0.074] | 0.002 | 0.006 |
| M2 (scalar) | 147.87 | 22 | 0.980 | 0.063 [0.053, 0.072] | 0.005 | 0.000 |
| Time 3 |  |  |  |  |  |  |
| M0 (configural) | 111.84 | 12 | 0.985 | 0.076 [0.063, 0.089] | — | — |
| M1 (metric) | 128.30 | 17 | 0.984 | 0.067 [0.057, 0.078] | 0.001 | 0.009 |
| M2 (scalar) | 149.21 | 22 | 0.981 | 0.063 [0.054, 0.073] | 0.003 | 0.004 |

Note. N = 2,915. All models were estimated using MLR. M0 (configural) = model for configural invariance; M1 (metric) = model for metric invariance; M2 (scalar) = model for scalar invariance. All models include within-time residual correlations for each subscale of MCER construct (rumination, self-blame, and catastrophizing) and cross-time autocorrelated residuals. *χ*^2^, chi-square statistic; *df*, degrees of freedom; CFI, comparative fit index; RMSEA, root mean square error of approximation; Δ values are calculated relative to the previous model.

**Table S5** Longitudinal Measurement Invariance of MCER Across T1–T3.

| Model | *χ^2^* | *df* | CFI | RMSEA [90% CI] | ∆CFI | ∆RMSEA |
| --- | --- | --- | --- | --- | --- | --- |
| M0 (configural) | 1108.01 | 105 | 0.963 | 0.057 [0.054, 0.060] | — | — |
| M1 (metric) | 1133.80 | 115 | 0.963 | 0.055 [0.052, 0.058] | 0.000 | 0.002 |
| M2(scalar) | 1230.74 | 125 | 0.959 | 0.055 [0.052, 0.058] | 0.004 | 0.000 |

Note. N = 2,915. MLR estimation. M0 (configural) = model for configural invariance; M1 (metric) = model for metric invariance; M2(scalar) = model for scalar invariance. All models include within-time residual correlations for each subscale of MCER construct (rumination, self-blame, and catastrophizing) and cross-time autocorrelated residuals. *χ*^2^, chi-square statistic; *df*, degrees of freedom; CFI, comparative fit index; RMSEA, root mean square error of approximation.

**SEM-Parallel Mediation**

To examine whether the subcomponents of maladaptive cognitive emotion regulation (MCER)—rumination, self-blame, and catastrophizing—operated differentially in the mediation model, a supplementary parallel mediation analysis was conducted. This analysis complements the second-order latent construct approach reported in the main text by allowing each MCER subcomponent to mediate independently.

Model fit: CFI = 0.947, RMSEA = 0.080, SRMR = 0.052, χ² = 613.28 (N = 2,915).

**Table S6** Mediation Analysis.

| **Path** | ***B*** | ***SE*** | ***β*** | **95% *CI*** | ***p*** | **Effect size** |
| --- | --- | --- | --- | --- | --- | --- |
| **Total effect** | 0.428 | 0.137 | 0.044 | [0.161, 0.696] | **0.002** | **-** |
| **Direct effect (Sex → D T3)** | 0.327 | 0.145 | 0.034 | [0.043, 0.611] | **0.024** | **-** |
| **Total indirect effect** | 0.101 | 0.061 | 0.011 | [-0.018, 0.221] | 0.097 | **-** |
| Sex → GP-NBE T1 → D T3 | -0.020 | 0.039 | -0.002 | [-0.096, 0.056] | 0.610 | 5.88% |
| Sex → GP-RA T1 → D T3 | -0.064 | 0.033 | -0.007 | [-0.128, -0.001] | **0.047** | 20.59% |
| Sex → SP-NSE T1 → D T3 | 0.059 | 0.049 | 0.006 | [-0.038, 0.155] | 0.232 | 17.65% |
| Sex → SP-WA T1 → D T3 | 0.120 | 0.050 | 0.012 | [0.023, 0.218] | **0.015** | 35.29% |
| Sex → T2 Rumination → D T3 | 0.005 | 0.011 | 0.001 | [-0.016, 0.026] | 0.637 | 2.94% |
| Sex → T2 Catastrophizing → D T3 | -0.005 | 0.006 | 0.000 | [-0.016, 0.007] | 0.420 | 0.00% |
| Sex → T2 Self-blame → D T3 | -0.046 | 0.018 | -0.005 | [-0.081, -0.011] | **0.010** | 14.71% |
| Sex → GP-NBE T1→ T2 Rumination → D T3 | -0.002 | 0.003 | 0.000 | [-0.008, 0.003] | 0.402 | 0.00% |
| Sex → GP-NBE T1→ T2 Catastrophizing → D T3 | 0.000 | 0.001 | 0.000 | [-0.002, 0.003] | 0.778 | 0.00% |
| Sex → GP-NBE T1→ T2 Self-blame → D T3 | 0.000 | 0.003 | 0.000 | [-0.005, 0.006] | 0.923 | 0.00% |
| Sex → GP-RA T1→ T2 Rumination → D T3 | 0.000 | 0.002 | 0.000 | [-0.004, 0.004] | 0.961 | 0.00% |
| Sex → GP-RA T1→ T2 Catastrophizing → D T3 | -0.003 | 0.002 | 0.000 | [-0.008, 0.002] | 0.206 | 0.00% |
| Sex → GP-RA T1→ T2 Self-blame → D T3 | -0.005 | 0.003 | 0.000 | [-0.010, 0.001] | 0.091 | 0.00% |
| Sex → SP-NSE T1→ T2 Rumination → D T3 | 0.012 | 0.005 | 0.001 | [0.002, 0.022] | **0.017** | 2.94% |
| Sex → SP-NSE T1→ T2 Catastrophizing → D T3 | 0.004 | 0.003 | 0.000 | [-0.002, 0.010] | 0.237 | 0.00% |
| Sex → SP-NSE T1→ T2 Self-blame → D T3 | 0.015 | 0.006 | 0.002 | [0.003, 0.027] | **0.013** | 5.88% |
| Sex → SP-WA T1→ T2 Rumination → D T3 | 0.011 | 0.005 | 0.001 | [0.001, 0.020] | **0.032** | 2.94% |
| Sex → SP-WA T1→ T2 Catastrophizing → D T3 | 0.010 | 0.007 | 0.001 | [-0.004, 0.025] | 0.162 | 2.94% |
| Sex → SP-WA T1→ T2 Self-blame → D T3 | 0.009 | 0.004 | 0.001 | [0.000, 0.018] | **0.042** | 2.94% |

*Note*. D = Depressive symptoms, B = unstandardized coefficient; β = standardized coefficient; SE = standard error; CI = confidence interval. The models were adjusted for significant background characteristics (i.e., age, school type, father’s and mother’s educational level, family structure, living arrangement, self-reported family financial status), adjusted for the indirect influence of sex on specific maladaptive cognitive emotion regulation strategies (i.e., Rumination, Catastrophizing, Self-blame) at T2 via corresponding variables at T1, and adjusted for the indirect influence of sex on depressive symptoms at T3 via depressive symptoms at T2. Following the methodological recommendations for models with suppression effects (i.e., the symbol of the specific indirect effect is opposite to that of the direct effect) (Wen & Ye, 2014), the effect sizes of shame/guilt and specific maladaptive cognitive emotion regulation strategies were calculated as the absolute ratio of the specific indirect effect to the direct effect, i.e., |ab/c'|×100%. The sum of individual indirect effect sizes may exceed 100% when suppression effects are present, as suppression and mediation effects operate in opposite directions.

**Figure S1** SEM of the mediation and suppression effects of shame/guilt proneness at T1 and specific MCER strategies at T2 between sex and depressive symptoms at T3.

*Note.* The value of sex (1 = male, 2 = female). Standardized coefficients were reported. The correlations of different variables at the same time are not presented in the model for simplification.

**p < .05, **p <.01, ***p <.001*

**Sensitivity Analysis-SEM Excluding the Low-Reliability SP-WA Subscale**

As the SP-WA subscale showed a low Cronbach alpha, a sensitivity SEM analysis was conducted by removing this subscale.

Model fit: CFI = .920, RMSEA = .065, SRMR = .054 (N = 2,915).

**Table S7** Mediation Analysis.

| Path | *B* | *SE* | 95% *CI* | *β* | *p* | Effect size |
| --- | --- | --- | --- | --- | --- | --- |
| **Total effect** | .408 | .136 | [.142, .674] | .043 | **.003** | - |
| **Direct effect** |  |  |  |  |  |  |
| Sex → D T3 | .386 | .137 | [.117, .656] | .040 | **.005** | - |
| **Indirect effect** | .022 | .048 | [-.072, .115] | .002 | .651 |  |
| Ind1: Sex → GP-NBE T1 → D T3 | -.021 | .039 | [-.097, .056] | -.002 | .596 | 5.00% |
| Ind2: Sex → GP-RA T1 → D T3 | -.059 | .033 | [-.124, .005] | -.006 | .070 | 15.00% |
| Ind3: Sex → SP-NSE T1 → D T3 | .086 | .047 | [-.007, .178] | .009 | .070 | 22.50% |
| Ind4: Sex → MCER T2 → D T3 | -.005 | .027 | [-.059, .048] | -.001 | .845 | 2.50% |
| Ind5: Sex → GP-NBE T1 → MCER T2 → D T3 | -.003 | .007 | [-.017, .011] | < .001 | .670 | 0.00% |
| Ind6: Sex → GP-RA T1 → MCER T2 → D T3 | -.009 | .006 | [-.021, .002] | -.001 | .111 | 2.50% |
| Ind7: Sex → SP-NSE T1 → MCER T2 → D T3 | .034 | .01 | [.014, .053] | .004 | **.001** | 10.00% |

*Note.* D = Depression, CI = Confidence interval. The models were adjusted for significant background characteristics (i.e., age, school type, father’s and mother’s educational level, family structure, living arrangement, self-reported family financial status), adjusted for the indirect influence of sex on MCER at T2 via MCER at T1, and adjusted for the indirect influence of sex on depression at T3 via depression at T2.

**Figure S2** SEM of the mediation and suppression effects of shame/guilt proneness (exclude the SP-WA subscale of shame proneness) at T1 and MCER at T2 between sex and depression at T3.

*Note.* MCER was created as a second-order latent variable defined by three first-order factors (rumination, self-blame, and catastrophizing). Standardized coefficients were reported. The correlations of different variables at the same time are not presented in the model for simplification.

*^†^p < .1, *p < .05, **p <.01, ***p <.001*

**Sensitivity Analysis-SEM with 2,727 Complete Cases**

A complete case analysis with a subsample of 2,727 participants who had no missing values on all three waves was conducted as a sensitivity check.

Model fit: CFI = .904, RMSEA = .062, SRMR = .066.

**Table S8** Mediation Analysis (*n* = 2,727).

| Path | *β* | *SE* | 95% *CI* | *p* | Effect size |
| --- | --- | --- | --- | --- | --- |
| **Total effect** | .094 | .029 | [.053, .138] | **.001** | - |
| **Direct effect** |  |  |  |  |  |
| Sex → D T3 | .059 | .022 | [.032, .103] | **.007** | - |
| **Indirect effect** | .036 | .014 | [.017, .064] | **.010** |  |
| Ind1: Sex → GP-NBE T1 → D T3 | -.014 | .007 | [-.027, -.004] | .055 | 23.73% |
| Ind2: Sex → GP-RA T1 → D T3 | -.011 | .005 | [-.021, -.005] | **.023** | 18.64% |
| Ind3: Sex → SP-NSE T1 → D T3 | .025 | .011 | [.009, .042] | **.020** | 26.60% |
| Ind4: Sex → SP-WA T1 → D T3 | .026 | .009 | [.015, .042] | **.003** | 27.66% |
| Ind5: Sex → MCER T2 → D T3 | -.003 | .007 | [-.022, .003] | .657 | 5.08% |
| Ind6: Sex → GP-NBE T1 → MCER T2 → D T3 | -.0004 | .002 | [-.004, .002] | .842 | .68% |
| Ind7: Sex → GP-RA T1 → MCER T2 → D T3 | -.004 | .002 | [-.009, -.001] | .075 | 6.78% |
| Ind8: Sex → SP-NSE T1 → MCER T2 → D T3 | .006 | .003 | [.002, .014] | **.040** | 6.38% |
| Ind9: Sex → SP-WA T1 → MCER T2 → D T3 | .011 | .004 | [.003, .018] | **.015** | 11.70% |

*Note.* D = Depressive symptoms, CI = Confidence interval. The models were adjusted for significant background characteristics (i.e., age, school type, father’s and mother’s educational level, family structure, living arrangement, self-reported family financial status), adjusted for the indirect influence of sex on MCER at T2 via MCER at T1, and adjusted for the indirect influence of sex on depressive symptoms at T3 via depressive symptoms at T2. Following the methodological recommendations for models with suppression effects (i.e., the symbol of the specific indirect effect is opposite to that of the direct effect) (Wen & Ye, 2014), the effect sizes of shame/guilt and MCER were calculated as the absolute ratio of the specific indirect effect to the direct effect, i.e., |ab/c'|×100%. The sum of individual indirect effect sizes may exceed 100% when suppression effects are present, as suppression and mediation effects operate in opposite directions.

**Figure S3** SEM of the mediation and suppression effects of shame/guilt proneness at T1 and MCER at T2 between sex and depressive symptoms at T3 (*N* = 2,727).

*Note.* The value of sex (1 = male, 2 = female). MCER was created as a second-order latent variable defined by three first-order factors (rumination, self-blame, and catastrophizing). Standardized coefficients were reported. The correlations of different variables at the same time are not presented in the model for simplification.

*^†^p < .1. *p < .05, **p <.01, ***p <.001*

**Reference**

**Chen, F. F. (2007).** Sensitivity of goodness of fit indexes to lack of measurement invariance. Structural Equation Modeling, 14(3), 464–504.

**Cheung, G. W., & Rensvold, R. B. (2002).** Evaluating goodness-of-fit indexes for testing measurement invariance. Structural Equation Modeling, 9(2), 233–255.

**Vandenberg, R. J., & Lance, C. E. (2000).** A review and synthesis of the measurement invariance literature: Suggestions, practices, and recommendations for organizational research. Organizational Research Methods, 3(1), 4–70.
